# Prevalence of Hepatitis B surface antigen (HBsAg) among visitors of Shashemene General Hospital voluntary counseling and testing center

**DOI:** 10.1186/1756-0500-4-35

**Published:** 2011-02-09

**Authors:** Asfaw Negero, Zufan Sisay, Girmay Medhin

**Affiliations:** 1 Department of Microbiology, Medewelabu University, Bale Goba, Ethiopia; 2 Aklilu Lemma Institute of Pathobiology, College of Health Sciences, Addis Ababa University, Addis Ababa, Ethiopia

## Abstract

**Background:**

Hepatitis B virus (HBV) infection is significant health problem, as it can lead to chronic hepatitis, liver cirrhosis, and hepatic carcinoma. Due to shared routes of transmission, HBV and human immunodeficiency virus (HIV) co-infection is common and is an emerging concern in the clinical management of patients because of increased mortality, accelerated hepatic disease progression, and the frequent hepatotoxicity caused by anti-retroviral therapy. The aim of this study was to determine the prevalence of Hepatitis B surface antigen (HBsAg) and its risk factors, among individuals visiting Shashemene General Hospital VCT center.

**Findings:**

Institution based cross-sectional study was performed from November 3, 2008 to December 29, 2008 and 384 voluntary counseling and testing (VCT) clients were investigated. Data on socio demographic and HBV risk factors was collected using structured questionnaires. Blood samples were collected and screened for hepatitis B surface antigen (HBsAg) and HIV by commercially available rapid test kits. The prevalence of HBsAg in this study group was 5.7%. Fourteen percent of HIV positive subjects (8/57) and 4.3% (14/327) of HIV negative subjects were positive for HBsAg. Significantly high prevalence of HBsAg was observed among individuals who had history of invasive procedures, like tooth extraction, abortion and ear piercing; history of hospital admission, history of unsafe inject and HIV positives.

**Conclusions:**

Although HBsAg prevalence is much higher among subjects who are HIV positive (14.0% versus 4.3%), the prevalence of HBsAg in HIV negative subjects is high enough to warrant a recommendation to screen all clients at VCT centers irrespective of HIV status.

## Background

Hepatitis B virus (HBV) infection is one of the major diseases of mankind that has shown to cause serious public health problem. It is estimated that about 2 billion people are infected with HBV world wide; of which more than 350 million have chronic HBV, and 1. 2 million die from chronic hepatitis, cirrhosis and hepatocellular carcinoma [1]. The global prevalence of HBV infection varies widely; and its endemicity ranges from high (≥8%) to intermediate (2-7%) and low (<2%) [2]. Regions like South East Asia and sub-Saharan Africa are high endemic areas [3] for HBV. Ethiopia, being part of this region, is ranked as an area with medium to high endemicity for HBV infection, based on previous population surveys [4,5].

In sub-Saharan Africa, almost all infections occur during the prenatal period or early childhood. Unscreened donated blood, unsafe therapeutic practices, including the use of inadequately sterilized needles and medical instruments [3], are the major routes of HBV transmission apart from sexual exposure in these regions. WHO estimate indicates that in developing counties using non-sterile, reused syringes or needles cause 12-18 million HBV infections annually [6].

HBV and HIV share common routes of transmission. In Ethiopia, heterosexual transmission is responsible for most HIV infections [7]. Additionally, Ethiopia being one of the countries most hit by HIV infection (prevalence of 2.1%) [8] and also found in a region classified as high endemic area for HBV [2]; the likelihood of HBV/HIV co infection is highly anticipated.

The consequences of HBV co infection with HIV is far reaching. In the United States, it is estimated that 10% of HIV infected individuals are HBV surface antigen positive [9] and that HIV infected individuals are 3 to 6 times more likely to develop chronic HBV infection than HIV uninfected individuals [10]. However, in Africa in general and in Ethiopia in particular, information on the magnitude of HBV positivity at different risk groups including people living with HIV/AIDS is scarce. Thus, studies to monitor exposure proportions and associated risk factors have paramount importance to undertake effective prevention measures. In line with this, Counseling and Testing, is a crucial intervention component of the HIV/AIDS prevention. Despite challenging circumstances, Ethiopia has dramatically increased its VCT coverage. The number of VCT facilities increased from 23 in 2001 to more than 1000 in 2007, and the number of HIV tests taken doubled in one year, from 1.7 million tests in 2007 to 3.5 million in 2008 [11].

Therefore, this study was conducted among clients of Shashemene General Hospital VCT center, to determine the prevalence of hepatitis-B surface antigen (HBsAg), co infection with HIV and associated risk factors.

## Methods

A hospital based cross-sectional study was conducted at Shashemene General Hospital, South Ethiopia, VCT center from November 3, 2008 to December 29, 2008. The VCT center at the hospital routinely gives HIV counseling and testing for the clients. Individuals tested positive for HIV infection were referred to anti-retroviral therapy (ART) clinics for further clinical and laboratory investigations and those found eligible for ART receive highly active anti-retroviral therapy (HAART). However, hepatitis testing was not performed in the hospital routinely.

The study population comprises of all individuals attending VCT center of Shashemene General Hospital for HIV testing. Counselor nurses interviewed the study subjects using structured questionnaire on socio-demographic characteristics and other risk factors.

Standard procedure was used during blood sample collection. Whole blood was used for HIV screening tests while serum was used for HBsAg screening. The hospital routinely uses commercial HIV test kits (KHB, Shangha Kehua Bio-engineering Co., Ltd. China) for screening and positive samples were re-tested with STAT-PACK (Chembio HIV 1/2 STAT-PAK™ Assay, CHEMBIO DIAGNOSTIC SYSTEMS, INC., MEDFORD, NY, USA). Samples giving discordant results in the two tests were re-examined using tie-breaker, (Uni-Gold HIV, Trinity Biotech PLC, Co. Wicklow, Ireland). All sera were screened for HBsAg using commercially available HBsAg screening rapid test kit according to the manufacturer's instruction (TULIP DIAGNOSTICS (P) LTD, India). The kit has a sensitivity of 87.3% and specificity of 100% [12].

Data were analyzed using SPSS version 15 soft ware. Proportions were used to summarize the frequency of occurrence of target outcomes in different categories of exposure variables. Precision of the prevalence of HBsAg across different categories of pre-specified risk factors was assessed by presenting 95% confidence interval around the estimates. Statistical significance of difference of proportion was evaluated using Chi-square test. P-value < 0.05 was considered as indicator for statistical significance.

The study was approved by the National Health Research Ethics Committee and Ethical Clearance Committee of Akililu Lemma Institute of Pathobiology. Separate permission was also obtained from Shashemene General Hospital. The purpose of the study was explained to the participants and written consent was obtained before sampling. HBs Ag positive participants were handled by the hospital physicians.

## Results

During the study period 391 individuals were approached and 384 were screened for HBsAg and HIV sero status resulting in 98.2% response rate. Socio-demographic characteristics of the participants are presented in Table 1. Of the total 384 VCT clients screened, 14.8% (95%CI: 11.4%, 18.8%) clients were sero positive for HIV and 5.7% (95%CI:3.6%, 8.5%) were positive for HBsAg. Fifty four percent of the participants were from Shashemene town and 52.1% were males resulting in the female to male ratio of 0.9:1. Among the study subjects 56.3% were married and 31.0% were never married, and 29.1% of the study subjects belonged to 30-39 years of age.

**Table 1:** Socio-demographic characteristics and proportion of hepatitis B surface antigen (HBsAg) among Different categories of study subjects at Shashemene General Hospital, 2008.

Characteristics	Total (%)	Number (%) positive HBsAg	chi-square value	P value
**Residence**				
Rural	178(46.4)	8(4.5)		
Urban	206(53.6)	14(6.8)	0.937	0.333
**Age**				
<20	79(20.5)	3(3.8)		
20-29	92(24.0)	5(5.4)		
30-39	112(29.2)	8(7.1)	2.923	0.570
40-49	57(14.8)	5(8.8)		
≥50	44(11.5)	1(2.3)		
**Sex**				
Male	200(52.1)	15(7.7)		
Female	184(47.9)	7(3.8)	2.423	0.120
**Marital Status**				
Married	216(56.3)	14(6.5)		
Single	119(31.0)	7(5.9)		
Divorced	12(3.1)	0	1.588	0.662
Widowed	37 (9.6)	1(2.7)		
**Religion**				
Christian	195(50.8)	11(5.6)	0.006	0.940
Muslim	189(49.2)	11(5.8)		
**Ethnicity**				
Oromo	183(47.7)	14(7.7)	2.639	0.451
Amhara	105(27.3)	5(4.8)		
Gurage	31(8.1)	1(3.2)		
Wolayta	65(16.9)	2(3.1)		
**Occupation**				
Merchant	96(25.0)	8(8.3)	3.381	0.760
House wife	92(24.0)	5(5.4)		
Student	44(11.5)	1(2.3)		
Farmer	63(16.4)	3(4.8)		
Driver	7(1.8)	1(14.3)		
Gov't Employee	18(4.7)	1(5.6)		
No work	64(16.7)	3(4.7)		
**Educational Status**				
Illiterate	142(37.0)	11(7.7)	5.238	0.155
Primary school	125(32.6)	5(4.0)		
Secondary school	l96 (25.0)	5(5.2)		
Diploma & above	21(5.5)	1(4.5)		

The distribution of hepatitis B surface antigen in different risk factors is indicated in Table 2. Of different risk factors considered in the study, history of having multiple sexual partners accounted the bigger number (34.6%; 95%CI: 29.9%, 39.6%). But the association of HBsAg and having multiple sexual partner was not statistically significant (P = 0.86). History of having invasive procedures (P = 0.006) and unsafe drug injection (injection of prescribed medication with unsteralized needles) (P **<**0.001) were significantly associated with increased risk of HBV carriage. Hospital admission was also significantly associated with HBsAg carriage (P = 0.015). Other risk factors, like blood transfusion, history of sexually transmitted infections (STIs), history of liver disease and contact with liver diseased family were not significantly associated with HBV carriage.

**Table 2:** Distribution of Hepatitis B virus (HBsAg) in subjects with different risk factors at Shashemene General Hospital, 2008.

Characteristics	Total (%)	Number (%) positive HBsAg	95% CI for HBsAg prevalence	chi-square value	P value
HBV vaccination					
Non vaccinated	377(98.2)	21(5.6)	3.5, 8.3	0.967	0.336
Vaccinated	7(1.8)	1(14.3)	0.3, 57.9		
History of Hospital admission					
No	369(96.1)	19(5.2)	3.1, 7.9	5.886	0.015
Yes	15(3.9)	3(20.0)	4.3, 48.1		
Unsafe inject					
No	370(96.4)	18(4.9)	2.9, 7.6		
Yes	14(3.6)	4(28.6)	8.4, 58.1	14.0368	<0.001
Multiple sexual partner					
No	251(65.4)	14(5.6)	3.1,9.2		
Yes	133(34.6)	8(6.0)	2.6,11.5	0.031	0.860
STIs					
No	376 (97.9)	21(5.6)	3.5,8.4		
Yes	8(2.1)	1(12.5)	0.3, 52.7	0.694	0.405
Invasive procedure*					
No	335 (87.2)	15(4.5)	2.5,7.3		
Yes	49 (12.8)	7 (14.3)	5.9,27.2	7.61	0.006
HIV status					
No	327(85.2)	14(4.3)	2.4,7.1		
Yes	57 (14.8)	8(14.0)	6.3,25.8	8.55	0.003

* abortion, tooth extraction, ear piercing, and tattooing

There were 8 clients with history of STIs and all of them were HIV positive and of 12 divorced individuals, 3 (25.0%) were HIV positive. Of total 22 HBs Ag positive individuals, 8(36.4%) were also positive for HIV and there was significant association between HBsAg carriage and HIV infection (P = 0.003).

## Discussion

In this hospital based cross-sectional study, a 5.7% prevalence of hepatitis B virus carriage among VCT clients of Shashemene General Hospital was determined. Similarly, a study conducted in Addis Ababa revealed a 5.7% HBsAg prevalence among VCT clients [13]. Another community based sero-epidemiological survey that addresses the transmission dynamics and control of hepatitis B virus in Addis Ababa, reported HBsAg prevalence of 7.0% from the general population [4], which is not significantly different from the current study (P = 0.373). A 3.7% HBsAg prevalence was also reported among pregnant women in Jimma [14].

In our study higher HBsAg positivity (14.0%) was observed among HIV positive individuals. This is comparable with a study done by Burnett *et al.*, [3] which documented an increased occurrence of HBV among HIV positive individuals. This could be due to the shared transmission route of both HBV and HIV infection. However, a study done from VCT centers in Addis Ababa reported no significant difference with HBsAg carriage and HIV serostatus [13]. Another study from the general population and from pregnant HIV infected women in the USA also indicated a much lower prevalence of HBV, 0.4% and 1.5%, respectively, as compared to the present study [15]. This could be due to the fact that in most developed parts of the world (Western Europe, USA, Australia), the endemicity of HBV infection is low [3].

In the present study no association has been found between HBsAg positivity and gender unlike other studies done in the country from VCT centers and general population, where male were found to have high HBsAg [4,13]. Similarly no association was observed with HBsAg positivity and other sociodemographic variables like, illiteracy and participants income. Nonetheless, a study done in Jimma among pregnant women has shown high HBsAg positivity rate among the illiterate and those with low income [14].

In agreement with other studies [2], in this study also significantly high prevalence of HBsAg was observed among individuals with history of invasive procedures, like tooth extraction, abortion [14], ear piercing and unsafe inject. In addition, participants with history of hospital admission had significantly high HBsAg (P = 0.015). This is comparable with study done in Greece in high-risk hospitalized patients [16]. Individuals who had multiple sexual partners were infected with HBV at higher rate, although the difference was not statistically significant.

This study is not without limitations. Our findings were based on rapid test kit which might have underestimated the true prevalence which could have been obtained if we were able to use radioimmunoassay antigen test or enzyme linked immunosorbent assay. Additionally, because of the small number of HBsAg positivity among study participants with history of known risk factors, we were not able to report the adjusted effects of these factors on the prevalence of HBsAg. This coupled with wide confidence interval of unadjusted effects suggest the importance of further study to establish the strength of the reported associations with these risk factors.

In conclusion high proportion of HBsAg was determined in this study. This indicates the importance of implementing preventive measures, including vaccination, to the health care workers at the VCT sites, as they are reported to be at increased risk of acquiring HBV compared to the general population [17]. In addition, the authors would also like to recommend the execution of routine HBV screening program at the hospitals VCT centers, especially for clients found positive of HIV antibody, by so doing HBV carriers could be identified early and managed appropriately. Moreover, awareness creation through health education about HBV transmission and prevention, including efforts to ensure all injections be administered with sterile syringes and needles is critical.

## Competing interests

The authors declare that they have no competing interests.

## Authors' contributions

ZS conceived the idea; ZS and AN designed the study; AN collected the sample, conducted laboratory work and drafted the manuscript; ZS supervised the overall conduct of the study and ZS, GM and AN interpreted the results; all authors participated in the write up; read and approved the final manuscript.
